# The Neural Dynamics of Conflict Adaptation within a Look-to-Do Transition

**DOI:** 10.1371/journal.pone.0057912

**Published:** 2013-02-28

**Authors:** Dandan Tang, Li Hu, Hong Li, Qinglin Zhang, Antao Chen

**Affiliations:** 1 Key Laboratory of Cognition and Personality of Ministry of Education, Faculty of Psychology, Southwest University, Chongqing, China; 2 Research Center of Psychological Development and Education, Liaoning Normal University, Dalian, Liaoning, China; University of Rome, Italy

## Abstract

**Background:**

For optimal performance in conflict situations, conflict adaptation (conflict detection and adjustment) is necessary. However, the neural dynamics of conflict adaptation is still unclear.

**Methods:**

In the present study, behavioral and electroencephalography (EEG) data were recorded from seventeen healthy participants during performance of a color-word Stroop task with a novel look-to-do transition. Within this transition, participants looked at the Stroop stimuli but no responses were required in the ‘look’ trials; or made manual responses to the Stroop stimuli in the ‘do’ trials.

**Results:**

In the ‘look’ trials, the amplitude modulation of N450 occurred exclusively in the right-frontal region. Subsequently, the amplitude modulation of sustained potential (SP) emerged in the posterior parietal and right-frontal regions. A significantly positive correlation between the modulation of reconfiguration in the ‘look’ trials and the behavioral conflict adaptation in the ‘do’ trials was observed. Specially, a stronger information flow from right-frontal region to posterior parietal region in the beta band was observed for incongruent condition than for congruent condition. In the ‘do’ trials, the conflict of ‘look’ trials enhanced the amplitude modulations of N450 in the right-frontal and posterior parietal regions, but decreased the amplitude modulations of SP in these regions. Uniquely, a stronger information flow from centro-parietal region to right-frontal region in the theta band was observed for iI condition than for cI condition.

**Conclusion:**

All these findings showed that top-down conflict adaptation is implemented by: (1) enhancing the sensitivity to conflict detection and the adaptation to conflict resolution; (2) modulating the effective connectivity between parietal region and right-frontal region.

## Introduction

Cognitive control is the basis of goal-directed behaviors, through which people can effectively utilize the limited cognitive resources to optimize the performance, especially when facing difficulty or interference [1], [2]. Conflict adaptation, often used to investigate the cognitive and neural mechanisms of cognitive control, refers to the conflict-driven sequential modulations of congruency effects in congruency task, e.g., Stroop task [3]. The congruency effects, indexed by the performance differences between incongruent and congruent conditions, are often smaller following incongruent compared to congruent condition [4]. Although conflict adaptation may be confounded by the antergic influence from bottom-up repetition priming [5], [6], as a top-down modulation, it will occur under appropriate experimental environments [7], [8], [9]. In terms of the popular conflict monitoring model [10], cognitive control consists of two basic components: evaluation of conflict occurrence and regulation of control, which have been universally acknowledged in the relevant studies [7], [11], [12], [13]. With regard to the neural architecture of cognitive control, three brain areas have been implicated: the anterior cingulate cortex (ACC), dorsal lateral prefrontal cortex (DLPFC), and posterior parietal cortex (PPC) (see [14] for an overall review).

However, there are some disputes concerning their particular functions in cognitive control. According to the conflict monitoring model [10], the ACC detects the occurrence of conflict, and then the DLPFC, which is activated by the signal from the ACC, implements a top-down adjustment to optimize performance [15], [16], [17], [18], [19]. In terms of the ACC-regulative account [20], the DLPFC evaluates conflict detection, and then the ACC regulates the control [21], [22]. Cognitive control studies also report modulations in the PPC [18], [23], [24], which has been shown to play a role in the resolution of stimulus-based conflict [7], [25] or motor preparatory activity [26]. Clearly, the two main accounts emphasize the role of the ACC, which can be corroborated by the findings from lesion studies which find evidence for impaired conflict adjustment in patients with ACC damage [19], [27]. However, other lesion studies in patients or animals indicate that the ACC is not specifically involved in interference processes, but is involved in motor preparation processes [28], [29], [30], [31]. Since lesion studies are able to demonstrate the necessity of one brain area for a particular cognitive function, the different results indicated that the role of the ACC in cognitive control is seriously questioned.

Since conflict adaptation refers to a sequential trial-to-trial modulation in the temporal course, event-related potentials (ERPs), which have millisecond resolution, can provide unique neural activation data for understanding this phenomenon in the time-domain studies. Some researches indicate that two ERP modulations primarily associate with the resolution of Stroop-type interference [3]: N450 and sustained potential (SP).

The N450 is a phasic negativity in the fronto-central region that reverses polarity in the fronto-lateral region, and is elicited about 400–550 ms following the presentation of a stimulus with non-response or response conflict [32], [33], [34], [35]. It may arise from the activity of a neural generator in the ACC [36] or anterior frontal cortex [21], [35], [37]. Recent research of conflict adaptation indicated that the N450 amplitude indexed current-trial congruency (greater amplitude for incongruent compared to congruent condition), but did not vary as a function of previous-trial congruency [38]. This finding suggests the amplitude modulations of N450 reflect conflict detection in the current trials [35], [39]. However, it is still unclear whether conflict detection is related to the fronto-central N450 or fronto-lateral N450.

The SP is a sustained parietal positivity or fronto-lateral negativity starting nearly 500 ms post-stimulus onset [37], [40]. The SP may be associated with general preparation [35], conflict processing [36], [37], [41], [42], or response selection [39]. Recent study found that the parietal SP amplitude not only indexed current-trial congruency (greater amplitude for incongruent compared to congruent condition), but also varied as a function of previous-trial congruency (greater amplitude for cI compared to iI condition) in the Stroop task. The result suggests that the amplitude modulations of parietal SP reflect conflict adaptation [38].

As a typical trial-to-trial regulation of cognition, conflict adaptation involves top-down information exchange among different brain areas (e.g., DLPFC, ACC, and PPC) [15]. However, the neural mechanisms of information exchange involved in conflict adaptation among these areas are not well understood. Recently, effective connectivity analysis [43] has been confirmed to provide a way to directly discuss the causal relationships between different brain areas, the results of which can reveal the basic mechanisms of high cognitive information communication [44]. The theoretical basis of it is the Granger causality [45]. Specifically, if previous values of X_1_ help to predict the future values of X_2_, a signal X_1_ is thought to cause a signal X_2_. Previously, the method based on the Granger causality has been applied to the study of cognitive information processing in the human brain [46], [47].

Traditionally, conflict adaptation is investigated within a do-to-do transition, where participants are required to execute a response in each trial (‘do’ trial). Specially, to execute a response in an incongruent trial, both conflict detection and resolution are necessary [48]. Since the intertrial intervals between these ‘do’ trials are normally short (e.g., range from 800 ms to 1,200 ms), the cortical processing evoked by the response execution in the previous ‘do’ trials will unavoidably impaired the trial-to-trial conflict adaptation in the current ‘do’ trials. Therefore, it is necessary to exclude the influence of response execution in the previous trials to investigate the unimpaired conflict adaptation in the current trials.

In the present study, we designed a novel look-to-do transition based on the Stroop task. Namely, ‘look’ trials were cued with asterisk (*) which informed participants just to look at the color of forthcoming word but not make any (overt or covert) response; ‘do’ trials were cued with cross (+) which informed participants to respond to the color of forthcoming word. Using the novel look-to-do transition design, the present study is able to examine unimpaired conflict adaptation when the proportion of congruent vs. incongruent trials is 50∶50 [49], [50], [51], [52], [53] and the neural dynamics of it. In terms of ERP modulations, we will focus on the amplitudes of N450 and SP both in the ‘look’ trials and ‘do’ trials. In addition, to demonstrate how the brain evaluates conflict and implements control, we examine the effective connectivity from right-frontal region to posterior parietal region for incongruent and congruent conditions in the ‘look’ trials, and from centro-parietal region to right-frontal region for iI and cI conditions in the ‘do’ trials, respectively.

## Materials and Methods

### Ethics Statement

Approval of the study was made by the Human Research Ethics Committee of the Southwest University of China, and all participants provided written informed consent.

### Participants

Seventeen self-report right-handed healthy undergraduates (9 females, aged from 20 to 24 years, 21.71±1.27, mean ± SD) with normal or corrected-to-normal vision and normal color perception took part in the study. They were paid for their participation. All were unaware of the purposes of the experiment.

### Stimuli

The stimuli consisted of four words RED, YELLOW, BLUE and GREEN [in Chinese, Song Ti No. 28 1.4° (horizontal) × 1.4° (vertical)], and were displayed in the center of a 17-in. screen using E-Prime software (Psychology Software Tools, Inc. Pittsburgh, PA). The viewing distance (between the participants and the computer screen) was approximately 60 cm. Responses were registered using a standard QWERTY keyboard. The words were colored in red, yellow, blue or green. The RGB values for the stimulus colors were 255, 0, 0 (red); 255, 255, 0 (yellow); 0, 0, 255 (blue); and 0, 255, 0 (green).

The trials were pseudo-random sequenced according to the congruency (congruent, incongruent) of ‘look’ trials and the congruency (congruent, incongruent) of ‘do’ trials. That resulted in an equal proportion of: cC trials (congruent ‘do’ trials preceded by congruent ‘look’ trials), cI trials (incongruent ‘do’ trials preceded by congruent ‘look’ trials), iC trials (congruent ‘do’ trials preceded by incongruent ‘look’ trials), and iI trials (incongruent ‘do’ trial preceded by incongruent ‘look’ trial) within the look-to-do transition. Trials, in which the ‘look’ trial was followed by the ‘do’ trial, were categorized in this way, whereas all other trials were discarded for the following analysis. All the transitions were based on two successive feature change trials where there were not (semantic) distractor-to-distractor, distractor-to-target (color), target-to-distractor, and target-to-target repetitions.

### Procedure and Task

**Figure 1:**
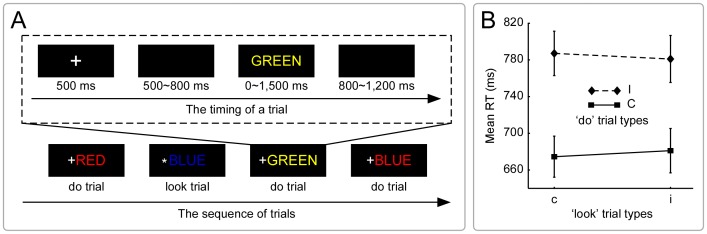
Experimental procedure and behavioral data. Panel **A** illustrates the timing parameters of one trial (above) and part of the trial sequence within the look-to-do transition design (below); the asterisk preceding the colored words informed participants only to look at the color of forthcoming word (‘look’ trial), the cross informed participants to respond to the color of the forthcoming stimulus (‘do’ trial). Panel **B** illustrates the mean RT in the ‘do’ trials as a function of congruency in the ‘look’ trials. The *RT_(iI–iC)_* was significantly smaller than *RT_(cI–cC)_* (error bars were SEM). NB. ‘c or C’ are the congruent condition; ‘i or I’ are the incongruent condition.

Fig. 1A depicts part of the trial sequence and the timing of one trial. All trials started with a fixation for 500 ms, followed by a blank screen for 500∼800 ms (interval varied randomly) from offset of fixation to target onset. A colored word was then presented until a key was pressed or for 1,500 ms, whichever came first. A blank screen was again presented for 800∼1,200 ms (interval varied randomly) from target offset to fixation onset, and then the next trial started. For each trial, the fixation was either an asterisk (*) or a cross (+), which indicated that the forthcoming trial would be a ‘look’ trial or a ‘do’ trial, respectively. In the ‘look’ trials, the participants were instructed to simply look at the color and ignore the semantics of words without executing any response. In the ‘do’ trials, the participants were instructed to press the “D” key using the left middle finger if the color of the word was red, the “F” key using the left forefinger if the color of the word was green, “J” key using the right forefinger if the color of the word was yellow, and the “K” key using the right middle finger if the color of the word was blue. They were instructed to perform the task as fast and as accurately as possible. Each participant completed one practice block including 64 trials prior to completing six experimental blocks including 870 trials, with a 2-min break between blocks. Since the present analyses focused on the look-to-do paired trials, i.e., each ‘look’ trial was followed by a ‘do’ trial, only the number of look-to-do paired trials was calculated. There were 320 ‘look’ trials and ‘do’ trials, respectively; the number of congruent and incongruent trials was equal. In addition, the ‘do’ trials were divided into cC, cI, iC, and iI conditions, each of which included 80 trials. The remainder-paired trials were excluded from the analyses.

### Electrophysiological Recording and Analyses

Electroencephalography (EEG) was recorded using a 64-channel system (Brain Products GmbH, Germany), with references on the left and right mastoids (average mastoid reference). The electrooculogram (EOG) was recorded with electrodes placed above and below the left eye. All interelectrode impedance was maintained below 5 kΩ during recording. The EEG and EOG were continuously sampled at 500 Hz with 0.01–100 Hz bandpass and the 50 Hz notch filter on. Trials contaminated with EOG artifacts (mean EOG voltage exceeding ±80 µV) or those with artifacts due to amplifier clipping, bursts of electromyographic (EMG) activity, or peak-to-peak deflection exceeding ±80 µV were excluded from averaging. ERP analysis epochs were extracted offline from −200 ms (pre-stimulus onset) to 1,000 ms (post-stimulus onset). ERPs were averaged for the ‘look’ trials where no response was executed, and for the ‘do’ trials where the responses were correct and response time (RT) was less than 1,500 ms.

Based on previous researches [32], [38], [40], [54], [55], [56] and the scalp topography distributions of the difference waves in the present study, the following scalp region-of-interests (ROIs) and time windows were defined. In the ‘look’ trials, we chose the left-frontal (F1, F3, FC1, FC3, and FC5), right-frontal (F2, F4, FC2, FC4, and FC6), fronto-central (C1, Cz, C2, and FC2), and posterior parietal (Pz, P2, P4, PO4, and POz) scalp regions, where the time windows of N450 (480–550 ms) and SP (700–800 ms and 800–900 ms) were respectively defined. In the ‘do’ trials, we chose the left-frontal (F1, F3, FC1, FC3, and FC5), right-frontal (F4, FC2, and FC4), fronto-central (FC2, FC4, Cz, and C2), and centro-parietal (P2, P1, POz, and CPz) scalp regions, where the time windows of N450 (400–450 ms) and SP (700–800 ms) were respectively defined.

In the ‘look’ trials, we compared the amplitudes of N450 and SP between incongruent and congruent conditions, respectively. To clarify whether ERP modulations in the ‘look’ trials predicted behavioral conflict adaptation in the post-‘look’ ‘do’ trials, we examined the correlations between the amplitudes of *N450_(I–C)_*, *SP_(I–C)_* in the ‘look’ trials and the *RT_(cI–cC)–(iI–iC)_* [57], [58], [59] in the post-‘look’ ‘do’ trials by conducting the Pearson’s correlation analysis (two-tailed) [60], [61]. Then, the casual relationship between right-frontal region and posterior parietal region was assessed using a time-varying effective connectivity [62], which is based on the concept of Granger causality [63]. This time-varying effective connectivity analysis was recently developed to capture fast changing information flows between neural activation from high-density EEG recordings [62]. The same analysis strategy, demonstrated in Hu et al [62], has been adopted in the present study. First, a time-varying multivariate autoregressive (tvMVAR) model was used to describe the evolution of single-trial variations of brain responses and a Kalman smoother was used to identify the tvMVAR model. The Kalman smoother had been proved to provide an accurate estimation of the tvMVAR coefficients. Second, the effective connectivity patterns (presented as time-varying partial directed coherence, tvPDC, in the time-frequency domain) were calculated from the Kalman smoother-based tvMVAR coefficient estimates [64] for both incongruent and congruent conditions. The significance of the tvPDC was subsequently evaluated using bootstrapping statistical analysis at the significance level of *p*<.01. The tvPDC values were evaluated from 1 to 30 Hz at a step of 1 Hz, and were baseline-corrected by subtracting and then dividing the average tvPDC values enclosed within the pre-stimulus reference interval (−150 ms to −50 ms) at each evaluated frequency.

In the ‘do’ trials, the two-way repeated-measure analyses of variance (ANOVAs) were first conducted for the mean amplitudes of N450 and SP with the following variables: the congruency of ‘look’ trials (congruent, incongruent) and the congruency of ‘do’ trials (congruent, incongruent). To assess the neural activities of conflict adaptation in the post-‘look’ ‘do’ trials, the amplitudes of N450*_(cI–cC)–(iI–iC)_* and SP*_(cI–cC)–(iI–iC)_* were calculated. Second, the casual relationship between right-frontal region and centro-parietal region was assessed using the time-varying effective connectivity for iI and cI conditions, the steps of which are same as what mentioned before. For the obtained PDC values, a paired sample *t*-test was conducted between iI condition and cI condition.

## Results

### Behavioral Data

Two two-way repeated-measure ANOVAs were respectively conducted with the following variables for the mean RT and error rates in the post-‘look’ ‘do’ trials: the congruency of ‘look’ trials (congruent, incongruent) and the congruency of ‘do’ trials (congruent, incongruent). Data of one participant was excluded because the overall accuracy was below 85%. The error trials (5% of ‘do’ trials), and the correct ‘do’ trials (0.94% of ‘do’ trials) where the participants falsely made responses in the pre-‘do’ ‘look’ trials were excluded from RT analysis.

For RT, the main effect of the congruency of ‘do’ trials was significant, *F*(1,15) = 66.88, *p*<.001, *η* ^2^ = .82. Importantly, the interaction between the congruency of ‘look’ trials and the congruency of ‘do’ trials was significant, *F*(1,15) = 4.90, *p*<.05, *η* ^2^ = .25 (Fig. 1B). To demonstrate the true conflict adaptation, post hoc tests were first conducted. However, the results did not reveal significant RT differences between iI condition and cI condition, nor between cC condition and iC condition, *ps* >.1. Then, the paired sample *t*-test was conducted. The key observation revealed that the *RT_(iI–iC)_* was significantly smaller than *RT_(cI–cC)_*, *t*(15) = −2.14, *p*<.05 (2-tailed). In addition, the one-sample *t*-test (2-tailed) was carried out to demonstrate whether the observed conflict adaptation [*RT_(cI–cC)–(iI–iC)_*] was significantly larger than the test value *0*. A significant conflict adaptation was found, *t*(15) = 2.14, *p* = .04. Accordingly, the RT patterns replicated and extended several previous observations, i.e., conflict adaptation was embodied in the conflict-driven decrease of congruency effects following incongruent relative to congruent ‘look’ trials [4], [65], [66], [67].

For error rates, the main effect of the congruency of ‘do’ trials reached significance, *F*(1,15) = 18.62, *p*<.001, *η* ^2^ = .55. However, the interaction between the congruency of ‘look’ trials and the congruency of ‘do’ trials was not significant, *F*(1,15) = 3.75, *p*>.05, *η* ^2^ = .20, the error rates were 3.56%, 5.56%, 3.44%, and 7.44% for cC, cI, iC, and iI trials, respectively; indicating that there was no trade-off between speed and accuracy.

### Electrophysiological Data

#### ERP results and effective connectivity analysis in the ‘look’ trials

**Figure 2:**
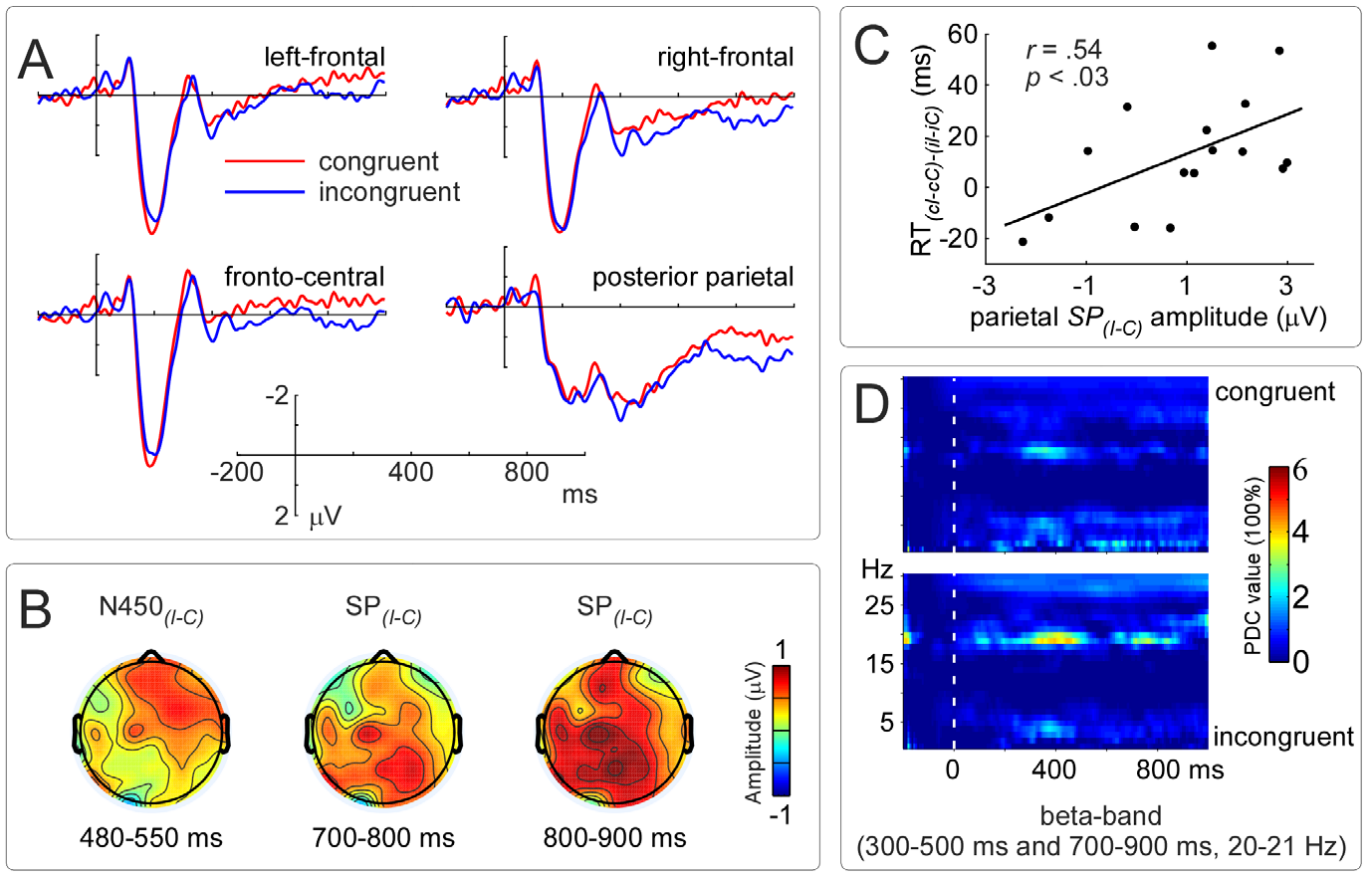
Electrophysiological results for the ‘look’ trials. Panel **A** illustrates the stimulus-locked grand-averaged ERP waveforms of incongruent and congruent conditions in the left-frontal (F1, F3, FC1, FC3, and FC5), right-frontal (F2, F4, FC2, FC4, and FC6), fronto-central (C1, Cz, C2, and FC2), and posterior parietal (Pz, P2, P4, POz, and PO4) scalp regions. Panel **B** illustrates the scalp topographies of the *N450_(I–C)_* and *SP_(I–C)_*. Panel **C** illustrates the significantly positive correlation between the amplitude of posterior parietal *SP_(I–C)_* (800–900 ms) in the ‘look’ trials and the *RT_(cI–cC)–(iI–iC)_* in the ‘do’ trials. Panel **D** illustrates the time-frequency distributions of the effective connectivity from right-frontal scalp region to posterior parietal scalp region, wherein incongruent condition showed stronger information flow in the beta band (20–21 Hz) in two distinct time windows (300–500 ms and 700–900 ms) compared with congruent condition. X-axis, time (ms); Y-axis, frequency (Hz). The white vertical bars indicate the stimulus onset. NB. ‘PDC’ is partial directed coherence.

The grand-averaged waveforms of incongruent and congruent conditions for the four ROIs are illustrated in Fig. 2A. The scalp topographies of difference wave for the N450 and SP are illustrated in Fig. 2B.

For the N450 (480–550 ms post-stimulus onset), the mean amplitude was more negative for congruent condition than for incongruent condition in the right-frontal region, *t*(15) = −2.27, *p*<.05; no significant differences were found in the other regions, *p*s >.05. For the SP, the mean amplitudes for congruent and incongruent conditions were significantly different: (1) from 700 ms to 800 ms in the posterior parietal region only, *t*(15) = −2.07, *p*<.05; (2) from 800 ms to 900 ms in the left-frontal region, *t*(15) = −2.05, *p*<.05; right-frontal region, *t*(15) = −1.97, *p*<.05; fronto-central region, *t*(15) = −2.23, *p*<.05; and posterior parietal region, *t*(15) = −2.32, *p*<.05.

We tested whether the amplitudes of *N450_(I–C),_ SP_(I–C)_* in the ‘look’ trials predicted the *RT_(cI–cC)–(iI–iC)_* in the post-‘look’ ‘do’ trials by calculating Pearson’s correlation (two-tailed). A significantly positive correlation between the amplitude of *SP_(I–C)_* (800–900 ms) in the posterior parietal region and *RT_(cI–cC)–(iI–iC)_*, *r* = .54, *p*<.03, was found (Fig. 2C). No other significant correlations were found, *p*s >.1.

Fig. 2D shows the time-frequency regions that exhibited significantly increased tvPDC values [68], in both incongruent and congruent conditions, as revealed using time-varying effective connectivity. To determine the information flow between the right-frontal and posterior parietal scalp regions, significantly increased tvPDC values [*p*<.01, false discovery rate (FDR) corrected, bootstrap analysis] were summarized via separation into two temporally distinct groups. In the two time windows (300–500 ms and 700–900 ms), significant increases in effective connectivity were observed from right-frontal scalp region to posterior parietal scalp region in beta-band (20–21 Hz) for incongruent condition (*p*<.02), but the connectivity was weaker (*p*>.05) for congruent condition.

#### ERP results and effective connectivity analysis in the ‘do’ trials

**Figure 3:**
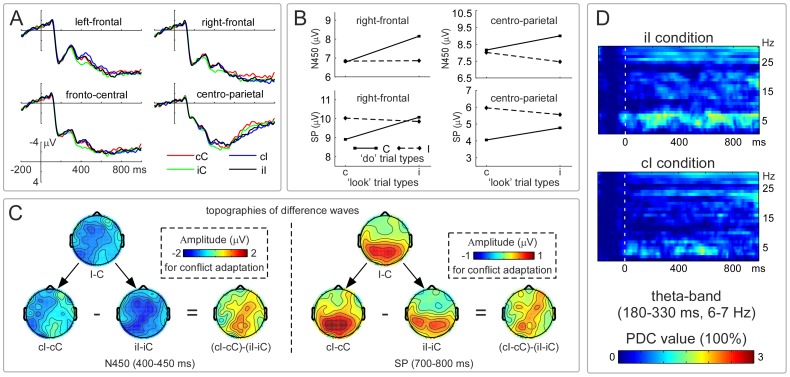
Electrophysiological results in the ‘do’ trials. Panel **A** illustrates the stimulus-locked grand-averaged ERP waveforms for cC, cI, iC, and iI conditions in the left-frontal (F1, F3, FC1, FC3, and FC5), right-frontal (F4, FC2, and FC4), fronto-central (FC2, FC4, Cz, and C2), and centro-parietal (P2, P1, POz, and CPz) scalp regions. Panel **B** illustrates the mean amplitudes of N450 (400–450 ms) and SP (700–800 ms) for cC, cI, iC, and iI conditions in the right-frontal and centro-parietal regions. Significant interactions between the congruency of ‘look’ trials and the congruency of ‘do’ trials are found for the amplitude modulations of both N450 and SP, which index neural adaptation. Panel **C** shows that the topographies of the *N450_(I–C)_* are different from those of the *SP_(I–C)_*. Although the activated topography distributions of *N450_(iI–iC)_* and *N450_(cI–cC)_* are contrasting with the *SP_(iI–iC)_* and *SP_(cI–cC)_*, those of the *SP_(cI–cC)–(iI–iC)_* and *N450_(cI–cC)–(iI–iC)_* are similar. The patterns suggest that the amplitude modulations of SP and N450 in the right-frontal and centro-parietal scalp regions reflect neural adaptation. Panel **D** illustrates the time-frequency distributions of the effective connectivity from centro-parietal scalp region to right-frontal scalp region for iI and cI conditions. X-axis, time (ms); Y-axis, frequency (Hz). Significant increase of effective connectivity from centro-parietal scalp region to right-frontal scalp region is observed in the theta band (180–330 ms, 6–7 Hz) for iI compared to cI condition. The white vertical bars indicate the stimulus onset. NB. ‘c or C’ are the congruent condition, ‘i or I’ are the incongruent condition; cI, cC, iI, and iC respectively refer to incongruent trials preceded by congruent trials, congruent trials preceded by congruent trials, incongruent trials preceded by incongruent trials, and incongruent trials preceded by congruent trials. ‘PDC’ is partial directed coherence.

The grand-averaged waveforms of cC, cI, iC, and iI trials for the four ROIs are illustrated in Fig. 3A. The mean amplitudes of the N450 and SP for the right-frontal and centro-parietal regions are illustrated in Fig. 3B. The scalp topographies of difference wave for the N450 and SP are illustrated in Fig. 3C. Table 1 illustrates the results of two-way repeated-measure ANOVAs to the mean amplitudes of N450 and SP for the four ROIs.

**Table 1:** The results of two-way repeated-measure ANOVAs to the mean amplitudes of N450 and SP in the ‘do’ trials.

The main effect of ‘look’ trial types												
	left-frontal			right-frontal			fronto-central			centro-parietal		
	*F*(1,15)	*p*	*η* ^2^	*F*(1,15)	*p*	*η* ^2^	*F*(1,15)	*p*	*η* ^2^	*F*(1,15)	*p*	*η* ^2^
N450	9.26	**	.38	5.18	*	.26	2.15	>.05	.12	<1	>.7	<.01
SP	1.33	>.05	.08	1.31	>.05	.08	<1	>.8	<.01	<1	>.7	<.01
**The main effect of ‘look’ trial types**												
	**left-frontal**			**right-frontal**			**fronto-central**			**centro-parietal**		
	*F*(1,15)	*p*	*η* ^2^	*F*(1,15)	*p*	*η* ^2^	*F*(1,15)	*p*	*η* ^2^	*F*(1,15)	*p*	*η* ^2^
N450	16.83	**	.53	3.31	*	.18	7.27	*	.34	9.11	**	.38
SP	<1	>.7	<.01	<1	>.7	.01	<1	>.8	<.01	6.03	*	.29
**The interaction between ‘look’ trial types and ‘do’ trial types**												
	**left-frontal**			**right-frontal**			**fronto-central**			**centro-parietal**		
	*F*(1,15)	*p*	*η* ^2^	*F*(1,15)	*p*	*η* ^2^	*F*(1,15)	*p*	*η* ^2^	*F*(1,15)	*p*	*η* ^2^
N450	2.49	>.05	.14	7.24	*	.33	1.57	>.05	.08	7.20	*	.32
SP	<1	>.7	<.01	3.57	*	.23	2.57	>.05	.15	3.59	*	.23
**Post hoc tests**												
	**right-frontal**						**centro-parietal**					
N450	cC<iC*			iI<cI, *p*>.05			iI<cI*			cC<iC, *p*>.05		
SP	cC<iC*			iI<cI, *p*>.05			iI<cI*			cC<iC, *p*>.05		

Note.*p<.05;

** *p*<.01. The time windows are 400–450 ms and 700–800 ms post-stimulus onset for the N450 and SP, respectively.

As displayed in Fig. 3C, the topographies of *N450_(cI–cC)–(iI–iC)_* (400–450 ms) and *SP_(cI–cC)–(iI–iC)_* (700–800 ms) revealed that conflict adaptation resulted in highly similar scalp activity distributions. Specifically, both the right-frontal and the centro-parietal regions were activated during the two time windows (400–450 ms, 700–800 ms). Those are consistent with the findings that significant amplitude interactions between the congruency of ‘look’ trials and the congruency of ‘do’ trials were found in these regions (Fig. 3B). Further analyses indicated that the congruency (congruent, incongruent) of ‘look’ trials led to contrasting influences on the N450 and SP. The amplitude of *N450_(iI–iC)_* compared to *N450_(cI–cC)_* showed stronger activation in the right-frontal and centro-parietal regions (Fig. 3C, left); however, the amplitude of *SP*_(*iI–iC)*_ compared to *SP*_(*cI–cC)*_ indicated weaker activation in these regions (Fig. 3C, right).

Fig. 3D illustrated the time-frequency regions that exhibited remarkable increase of tvPDC values (FDR corrected) for iI condition than for cI condition. A significant increase in effective connectivity was observed from centro-parietal scalp region to right-frontal scalp region in the theta-band (180–330 ms, 6–7 Hz) for iI condition than for cI condition, *t*(15) = 2.17, *p*<.05. However, the connectivity was comparable in the theta-band (600–1,000 ms, 6–7 Hz) between iI and cI conditions, *t*(15) = 1.58, *p*>.1.

## Discussion

In a standard Stroop task where the proportion of congruent trials is equal to that of incongruent trials, we found significant neural and behavioral conflict adaptation within a novel look-to-do transition design. The RT pattern of conflict adaptation (Fig. 1B) was manifested in smaller *RT_(iI–iC)_* compared with *RT_(cI–cC)_*, which was consistent with the previous observations [4], [65], [66], [67]. The electrophysiological data of present study revealed intriguing neural dynamics of conflict detection and control implementation. In the ‘look’ trials, the activity of amplitude of *N450_(I–C)_* implicated the right-frontal region; but that of *SP_(I–C)_* implicated the right-frontal, fronto-central, and posterior parietal regions (Fig. 2A and 2B). In the ‘do’ trials, the activity of amplitude of *N450_(I–C)_* implicated the left-frontal, right-frontal, fronto-central, and centro-parietal regions, but that of *SP_(I–C)_* was limited to the centro-parietal region (Fig. 3A and 3C). Specially, effective connectivity revealed that the cortical information was consistently flowed from right-frontal region to posterior parietal region in the beta band in the ‘look’ trials (Fig. 2D), and from centro-parietal region to right-frontal region in the theta band in the ‘do’ trials (Fig. 3D). All these results suggest that conflict adaptation mediates the cortical processing involving in the interaction of multiple functionally specialized cortical regions.

In the ‘look’ trials, the right-frontal N450 was a reverse polarity N450 (more negative amplitudes in congruent compared to incongruent condition), which was also found in the fronto-lateral regions by West and colleagues [33], [35]. Since the N450 has been related to conflict detection, primarily observed in Stroop-type conflict [35], [39], the reverse polarity N450 should reflect conflict detection in the ‘look’ trials. Besides, since the response executions which will result in strong activation in the fronto-central region, such as the ACC [30], have been excluded in the ‘look’ trials, the conflict detection has not been contaminated by them in these trials. Therefore, the reverse polarity N450 may reflect that the brain recruits more resources to process colors when facing the interruption from incongruent color-word codes in incongruent condition than in congruent condition. Indeed, evidence from patients with ACC damage [28] and monkeys with ACC lesions [29] show the intact conflict adjustment. However, it disappears entirely following DLPFC lesions [29]. These results indicate that the DLPFC plays a critical role in conflict adjustment.

The amplitude modulation of SP in the posterior parietal region started at 700 ms post-stimulus onset. The latency difference between N450 and SP may reflect the minimal time requirement (about 200 ms) between conflict detection and reconfiguration of the cognitive system for conflict adjustment [69], [70]. Previous studies have indicated that the amplitude modulation of SP in the posterior parietal region was associated with conflict adaptation [38], [55], [61] or response selection [39]. Whereas there is no response execution in the ‘look’ trials, we propose that the amplitude modulation of posterior parietal SP may reflect the processing of information reconfiguration or control preparation [35].

In general, a activated brain area will favor its function execution in completing the forthcoming task [15]. In fact, the significantly positive correlation between the amplitude of *SP_(I–C)_* (800–900 ms) in the posterior parietal region in the ‘look’ trials and the *RT_(cI–cC)–(iI–iC)_* in the ‘do’ trials (Fig. 2D) suggested that the larger amplitude of *SP_(I–C)_* predicts the stronger conflict adaptation. Since the PPC has been shown to play a role in the resolution of stimulus-based conflict [7], [25] or motor preparatory activity [26], the amplitude modulation of SP in the posterior parietal region may reflect the resolutions of stimulus conflict or imaginary response conflict. In addition, the amplitude modulations of SP (800–900 ms) in the right-frontal region indicate that the conflict information may be maintained in the DLPFC [29].

About in the N450 and SP time windows in the ‘look’ trials, the effective connectivity results showed a stronger cortical information flow from right-frontal scalp region to posterior parietal scalp region in the beta band (20–21 Hz, 300–500 ms and 700–900 ms) for incongruent than for congruent condition (Fig. 2C). Although the spatial resolution limitations of using EEG/ERP methods, it suggests that the information related to conflict detection may have been effectively transferred from right-frontal region to posterior parietal region. These results can be explained by the proposes that the beta-band modulation supports the maintenance of the current sensorimotor or cognitive state [71].

In the ‘do’ trials, the interaction between the congruency of ‘look’ trials and the congruency of ‘do’ trials for N450 amplitude was significant in the right-frontal and centro-parietal regions (Fig. 3B). The topographies showed that larger amplitude for *N450_(iI–iC)_* than for *N450_(cI–cC)_* were evoked in these regions; but the left-frontal or fronto-central regions were not affected by the incongruent-‘look’ context. Furthermore, the topography clearly indicated the right-frontal and centro-parietal distributions of *N450_(cI–cC)–(iI–iC)_* (Fig. 3C). Recent studies in healthy human and individuals with mild and moderate-to-severe traumatic brain injury [38], [50], [54], [55], [72], [73] also did not show conflict adaptation for the fronto-central N450. The present study found that top-down conflict adaptation was embodied in the amplitude modulations of N450 in the right-frontal and centro-parietal regions, which corroborated and expanded the previous studies. Thus, we think that the larger *N450_(iI–iC)_* amplitude suggests that the conflict of ‘look’ trials may have enhanced the sensitivity of the brain to the conflict occurrence. In addition, the latency of N450 was shorter in the ‘do’ trials than in the ‘look’ trials, maybe implying that the conflict detection is easier and earlier in the ‘do’ trials.

Interestingly, in the ‘do’ trials, the interaction between the congruency of ‘look’ trials and the congruency of ‘do’ trials for SP amplitude was significant in the right-frontal and centro-parietal regions (Fig. 3B). Furthermore, the topography distribution of *SP_(cI–cC)–(iI–iC)_* mainly implicated the right-frontal and centro-parietal regions (Fig. 3C, right), which was driven by stronger *SP_(cI–cC)_* compared to *SP_(iI–iC)_* activation in the centro-parietal region. In the literature, the SP has been associated with multiple functions, including general preparation [35], conflict processing [36], [37], [41], [42], response selection [39], and conflict adaptation [38]. The focused activation in the centro-parietal region indicates that executing responses in the ‘do’ trials may need the brain to recruit most of cognitive resources to conduct the response-related processing (e.g., response selection or response-conflict resolution). Accordingly, we suggest that the amplitude modulations of SP in the ‘do’ trials are affected by the ‘look’-trial congruency, and therefore reflect conflict adaptation.

It is worth noting that there was no significant RT difference between iI and cI conditions in the ‘do’ trials. However, as revealed by effective connectivity in the ‘do’ trials, the cortical information is more consistently flowed from centro-parietal scalp region to right-frontal scalp region in the theta band (6–7 Hz, 180–330 ms) for iI compared to cI condition. This performance-invariant neural difference in effective connectivity may reflect the basic neural mechanisms of conflict adaptation. Namely, the evaluation of conflict occurrence and regulation of control can be reflected in the theta-band modulation in human brain. Therefore, the present result provides novel evidence for understanding the course of information exchange between different brain areas related to conflict detection and potential conflict resolution in cognitive control.

In conclusion, using the novel look-to-do transition design, the profile of conflict adaptation can be clearly determined. In the ‘look’ trials, the amplitude modulation of N450 (480–550 ms) in the right-frontal region reflects conflict detection. A more effective information flow is transferred from right-frontal region to posterior parietal region in incongruent compared to congruent condition. The amplitude modulation of SP appears in the posterior parietal region (700–800 ms), frontal- and parietal- regions (800–900 ms). In the post-‘look’ ‘do’ trials, conflict adaptation is reflected in the amplitude modulations of N450 and SP in the right-frontal and parietal regions. Moreover, a more effective information flow is transferred from centro-parietal region to right-frontal region for iI compared to cI condition. The present findings reveal that conflict adaptation mediates the cortical processing involving in the interaction of multiple functionally specialized cortical regions by enhancing the sensitivity to conflict detection and the adaptation to conflict resolution.
